# Hydrogen Gas Phase and Electrochemical Hydriding of LaNi_5−x_M_x_ (M = Sn, Co, Al) Alloys

**DOI:** 10.3390/ma14010014

**Published:** 2020-12-22

**Authors:** Stanislava Todorova, Borislav Abrashev, Vesselina Rangelova, Lyuben Mihaylov, Evelina Vassileva, Konstantin Petrov, Tony Spassov

**Affiliations:** 1 Faculty of Chemistry and Pharmacy, Sofia University “St. Kl. Ohridski”, 1 James Bourchier Blvd., 1164 Sofia, Bulgaria; nhtst@chem.uni-sofia.bg (S.T.); nhtvr@chem.uni-sofia.bg (V.R.); nhtlm@chem.uni-sofia.bg (L.M.); ejvasileva@uni-sofia.bg (E.V.); 2 Institute of Electrochemistry and Energy Systems, Acad. Evgeni Budevski, Bulgarian Academy of Sciences, Acad. G. Bonchev Str., Bl. 10, 1113 Sofia, Bulgaria; babrashev@iees.bas.bg (B.A.); k.petrov@iees.bas.bg (K.P.)

**Keywords:** LaNi_5_ alloys, hydrogen capacity, hydriding kinetics, cycle life, Ni–MH batteries

## Abstract

Hydriding/dehydriding properties of a series of LaNi_5_ based alloys were compared by applying both hydrogen gas phase and electrochemical hydrogen charge/discharge methods. The highest hydrogen absorption capacity of 1.4 wt.% H_2_ was found for LaNi_4.3_Co_0.4_Al_0.3_, although LaNi_4.8_Sn_0.2_ also reveals comparable hydrogen capacity (>1.3%). A significant difference in the hydriding kinetics was observed for all studied alloys before and after activation. The activated alloys (5 cycles at 65 °C, 40 atm. H_2_) reach their maximum capacities after less than a minute, whereas the pure LaNi_5_ alloy needs several minutes for complete hydriding. The electrochemical hydriding/dehydriding behavior of the alloys reveals superior performance of LaNi_4.3_Co_0.4_Al_0.3_ and LaNi_4.8_Sn_0.2_ compared to the other compositions studied, as the capacity of LaNi_4.8_Sn_0.2_ decreases by only 10% for 60 charge/discharge cycles at a current density of 100 mA/g. Good agreement between the hydrogen sorption kinetics of the alloys obtained electrochemically and from hydrogen gas phase has also been observed.

## 1. Introduction

Intermetallic compounds based on LaNi_5_ are still a fundamental and applied research interest, as they can be used as materials for hydrogen storage both in gas storage tanks and as anodes in Ni-MH batteries [1,2,3]. The performance of such devices is strongly dependent on the reversible storage capacity of the material used as storage media. The main drawback of alloys based on LaNi_5_ is their low hydrogen storage density, which is the reason why Li-ion batteries became dominant over the last years in portable electronic devices and rechargeable batteries for electric and hybrid vehicles. Despite this, researchers are still interested in anode material improvement because Ni–MH batteries have lower prices, high charge/discharge rates and are safer in regards to overheating [4,5].

A popular approach for improvement of LaNi_5_ performance is a substitution of La by Mm and Ni by different metals like Al, Cr, Mn, Co, Fe, Sn, Cu, etc. The substitution of La by Mm contributes most to the price of the final product without compromising the electrochemical characteristics, while partial substitution of Ni in the crystal lattice was shown to significantly improve the performance of the alloy [5]. Co, Al and Mn are known to increase the cell volume, decrease the volume expansion upon hydriding and decrease the corrosion rate, thus leading to higher electrochemical capacity and improved cycle life [6,7]. These elements are the most commonly used ones for substitution of Ni in commercial alloys for Ni–MH batteries [8]. In gas storage, Co, Al and Mn have a positive effect as well—the increased unit cell volume leads to decreased plateau pressure and increased hydride stability [9,10,11]. Another element that is widely used as a substitute for Ni and has a very good effect of hydrogen storage properties of LaNi_5_ is Sn. Typical alloys that have been studied are represented by the general formula LaNi_4−x_Sn_x_, where x varies in the range 0 ≤ x ≤ 0.5 [12,13,14,15]. The effect of tin addition was first evaluated for gas storage, showing that the prepared alloys have a larger unit cell volume compared to LaNi_5_ and a significant decrease of the plateau pressure which enhanced hydrogen storage properties [13,14,15]. It was also noted that improved stability upon an absorption—desorption cycling is proportional to the extent of Ni substitution up to x ≤ 0.4 [14,15]. Electrochemical cycling of Sn substituted alloys shows that the discharge capacity is significantly improved, reaching a maximum value of 300 mA/g in case of LaNi_4.58_Sn_0.42_. However, electrochemical discharge values do not show a linear correlation with the Sn content and the discharge capacity values strongly depend on the charge/discharge conditions [12,16].

In recent years, most relevant research studies have on one hand concentrated on the investigation of discharge capacity value performances at elevated temperatures or after thousands of cycles [17] and on the other on a thermodynamic characteristic like ternary or quaternary phase diagrams of the metals included in the alloy and hydrogen [18]. The hydrogen diffusion coefficient is another thermodynamic characteristic that is still of interest and has been studied through different techniques—nuclear magnetic resonance, various electrochemical techniques, neutron scattering or a combination of these [19,20]. Therefore in the present study, we concentrated on the complex characterization of hydrogen capacity and kinetics, both from a hydrogen gas phase as well as electrochemically, for the following alloys for comparison: LaNi_4.3_Co_0.4_Al_0.3_, LaNi_4.8_Sn_0.2_, LaNi_4.7_Sn_0.3_ and LaNi_5_. The electrochemical performance of LaNi_4.3_Co_0.4_Al_0.3_ was previously studied by K. Giza [21] and showed a high capacity of 310 mA/g. However, there are no data about the electrochemical behavior that occurs during more than 10 cycles and there are no data for the gas phase hydrogenation. It is also of interest to compare the performance of LaNi_4.3_Co_0.4_Al_0.3_ to that of Sn substituted alloys, which are known for their very good resistance to cycling both from a gas phase and electrochemically [12,14,15,22,23,24,25]. It was shown that optimal content of Sn in LaNi_5−x_Sn_x_ is in the range 0.2 ≤ x ≤ 0.3 [24]. In that range, the hydrogenation properties of the alloys have significant differences [12,14]. Therefore, the aim of the present study was to compare them both electrochemically and from a gas phase; moreover, the aim was also to assess alloys with Sn content of exactly 0.3, which have been less commonly studied. The hydrogen diffusion coefficients for these compositions were also determined using potentiostatic discharge conditions and compared to the rather scattered existing values in the literature.

## 2. Materials and Methods

LaNi_5_ based alloys (LaNi_5_, LaNi_4.3_Co_0.4_Al_0.3_, LaNi_4.8_Sn_0.2_ and LaNi_4.7_Sn_0.3_) were synthesized by induction melting of the pure metals (purity 99.999%), followed by re-melting to achieve chemical homogeneity of the alloys. The amount of the ingots was about 100 g and the melting was realized under vacuum by induction heating at a temperature of 1350–1400 °C. Then the alloys were pulverized by hydriding at 50 °C under 50 bar pure hydrogen atmosphere.

The structure and microstructure of the alloys were studied using X-ray diffraction (XRD) with Cu-K_α_ radiation and the morphology and size of the powders particles were characterized using scanning electron microscope JEOL 5510 (Jeol Ltd., Tokyo, Japan). ImageJ 1.53b software was applied to obtain the particles size distribution.

Hydrogen sorption kinetic curves of the samples were measured by a home-made Sieverts’-type apparatus at room temperature (20 °C maintained by thermostat and at a constant pressure of 10 and 40 atm H_2_). The total volume of the device was 0.35 L and the sample weight was about 300 mg. The precision of the pressure sensor was 0.01%. The kinetic curves presented in the study were obtained after the five initial hydriding/dehydriding cycles (65 °C, 40 atm H_2_) required for the alloys activation.

The electrochemical behavior of the alloys during charge/discharge cycling was studied using a three electrode cell. The working electrode was prepared using 100 mg of the synthesized materials, 70 mg of teflonized carbon and 0.5 mL of heptane. The mixture was pressed at about 150 atm. to form the electrode and then dried in air. NiOOH/Ni(OH)_2_ was used as a counter electrode and Ag/AgCl was used as a reference. Each electrode was charged for 6 h at 5 mA and discharged to 500 mV at 2 mA in a water solution of 6 mol/dm^3^ KOH (Sigma Aldrich, Product number 30603). Additionally, a higher current density of 10 mA of charge and discharge was applied during the cycling stability test for all alloys.

To determine the hydrogen diffusion coefficient in the LaNi_5_ alloys, first the samples were fully hydrided under galvanostatic conditions. Then, the hydrogen charged samples were discharged at potentiostatic conditions (900 mV) in the same electrolyte. The discharge potential was selected to correspond to the discharge potential plateau from the galvanostatic charge/discharge experiments.

## 3. Results and Discussion

### 3.1. Alloys Morphology and Microstructure

**Figure 1:**
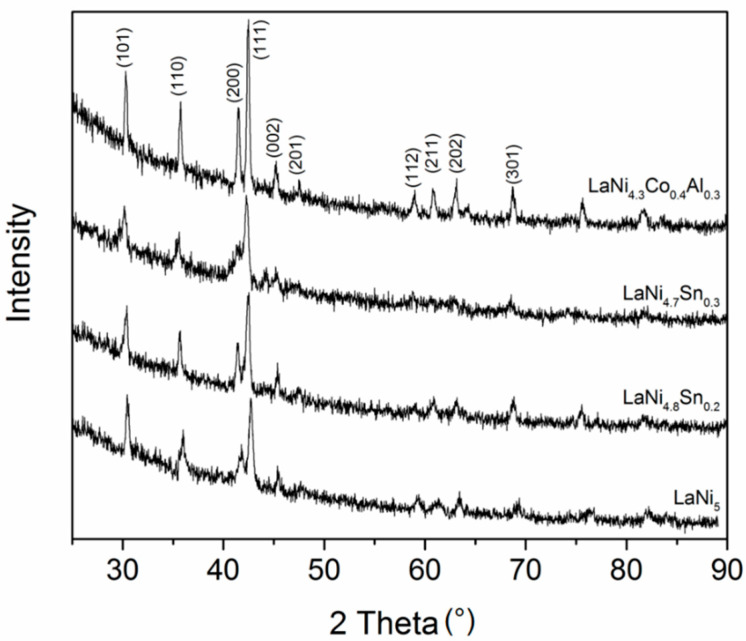
X-ray diffraction (XRD) patterns of the studied LaNi_5_ based alloys (Cu-Kα radiation).

X-ray diffraction analysis revealed the same hexagonal CaCu_5_ type structure of all LaNi_5_ based alloys studied, as shown in Figure 1. The shift of the diffraction peaks maximums to lower diffraction angles was due to the alloying and was more pronounced when Ni was partially substituted for by Sn, due to the larger size of the tin atom compared to the nickel atom. Table 1 shows the XRD results for the alloys, including the lattice constants, cell volume and Atomic Radius Factor (calculated according to [26]). It was seen that the crystal lattice volume increases with the addition of all metals substituting for Ni in LaNi_5_. Judging by the width and intensity of the diffraction peaks, the alloys that were assessed also have a similar microstructure (crystallite size, strain) except for LaNi_4.7_Sn_0.3_, which is characterized with noticeably finer crystallites that have an average size of about 35 nm (according to Scherrer’s method).

**Figure 2:**
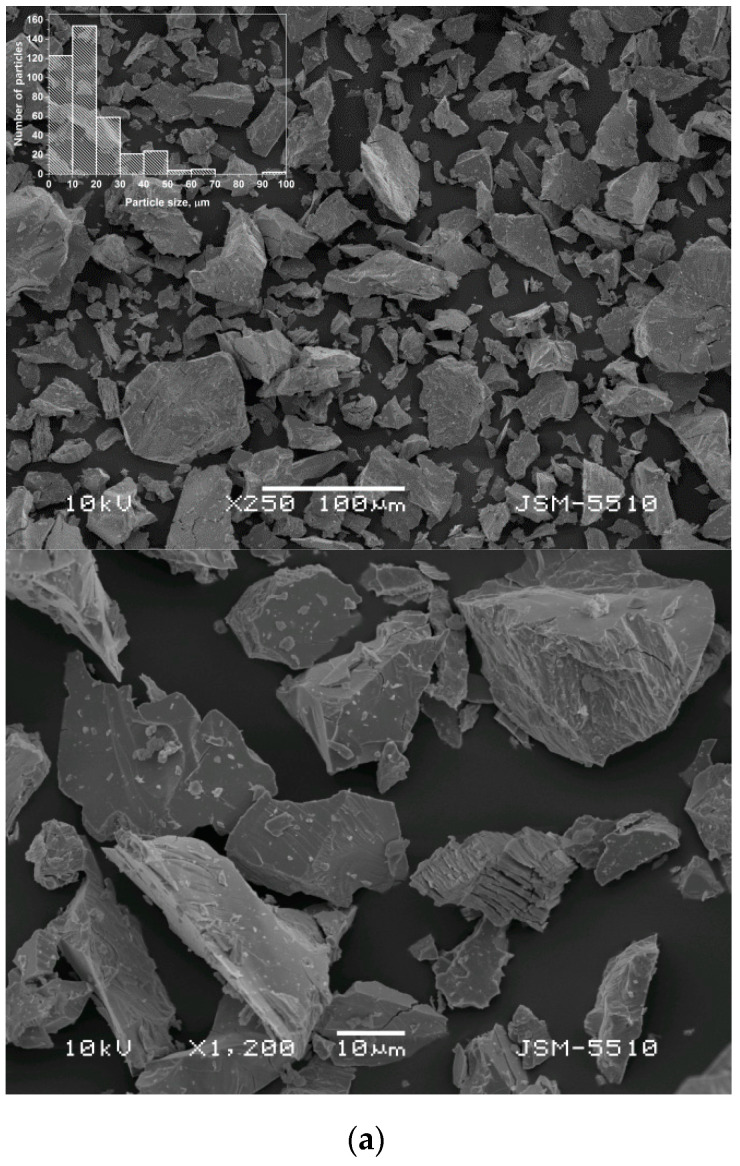

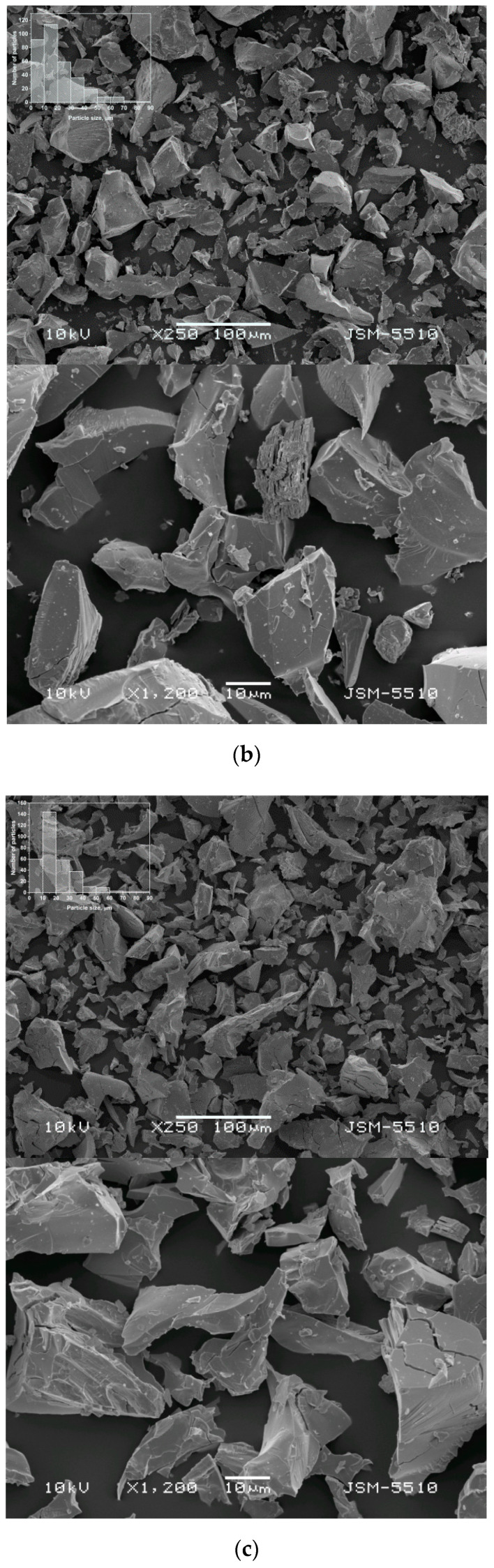

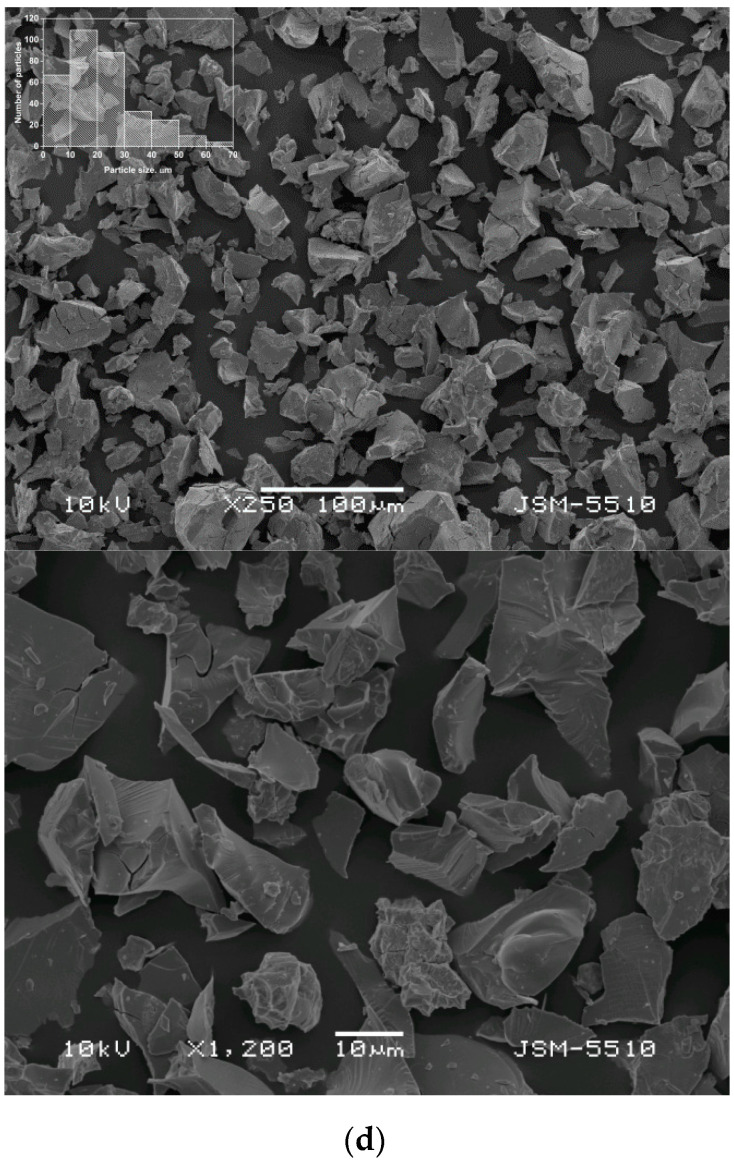
Scanning electron microscopy micrographs of LaNi_5_ based alloys and particle size distribution histograms, (**a**) LaNi_5_; (**b**) LaNi_4.3_Co_0.4_Al_0.3_; (**c**) LaNi_4.8_Sn_0.2_; (**d**) LaNi_4.3_Sn_0.3._

The alloys’ powders did not show any major impacts on the particles’ morphology and sizes as well, as shown in Figure 2. Particles with an average size of 18–20 μm and an irregular shape occurred for all of the compositions that were assessed. The size distributions were relatively broad, including particles varying from several micrometers to 100 μm, as shown in Figure 2 (insets). At higher magnifications, particle cracks were also clearly observed. The cracks could be better detected on the larger particles (>100 μm), which obviously failed to fully fragment during the initial pulverization treatment (which is described in the experimental section). However, such particles were not commonly found in the powder samples and needed to be sought for this purpose.

### 3.2. Hydrogen Gas Phase Absorption

**Figure 3:**
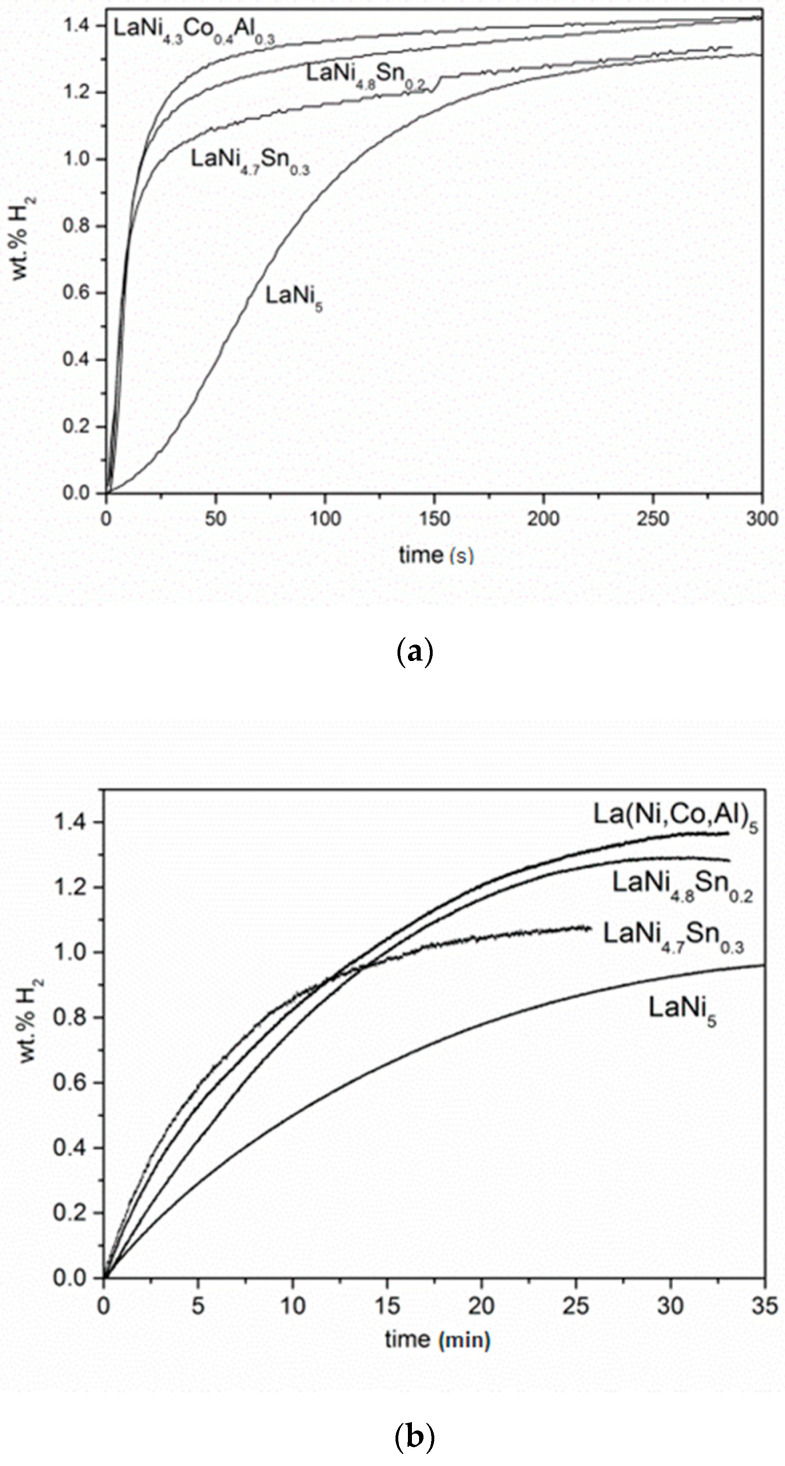
Hydrogen absorption kinetic curves of the activated LaNi_5_ based alloys (20 °C, 10 atm) (**a**); and before activation (**b**).

Initially, the hydrogen sorption properties of the alloys (LaNi_4.3_Co_0.4_Al_0.3_, LaNi_4.8_Sn_0.2_, LaNi_4.7_Sn_0.3_) were studied during a hydrogen gas phase and were compared to pure LaNi_5_. Figure 3 shows hydrogen absorption curves at a pressure of 10 atm. and at room temperature (20 °C) after initial activation of the alloys (5 absorption/desorption cycles at 65 °C). In practice under these conditions, all of the studied compositions, except for LaNi_5_, reached their maximum capacity after less than a minute. The absorption kinetic curves were fully reproducible after the initial alloys activation. The maximum hydriding capacity of a given alloy measured in 3 consecutive absorption experiments differed in the range of only +/− 5%. However, it is necessary to point out that the same alloys revealed much slower absorption kinetics prior to activation (see Figure 3b). It is commonly known that generally, the creation of a high density of defects such as planar defects (stacking faults), dislocations, point defects or compositional fluctuations [27], is the major mechanism for the activation of the hydriding process in hydrogen storage alloys. For LaNi_5_, it was proven that after the initial activation cycles, the creation of planar defects in the alloy enhances hydrogen diffusion and assists with the subsequent transition of the α-phase to the hydride on later hydriding cycles.

It is obvious that compared to the binary LaNi_5_, all alloys in the present study revealed substantial improvement of the hydrogen sorption kinetics and capacity. The LaNi_4.3_Co_0.4_Al_0.3_ alloy achieved maximum capacity (1.4 wt.% hydrogen) faster than the other alloys, followed by LaNi_4.8_Sn_0.2_ and LaNi_4.7_Sn_0.3_. Similar hydrogen absorption capacities, decreasing from 1.35 wt.% to 0.95 wt.% with an increasing Sn content, were obtained by other authors for LaNi_5−x_Sn_x_ (0 ≤ x ≤ 0.5) alloys [28]. The increased observed hydrogen storage capacity of the alloys compared to pure LaNi_5_ was an expected consequence of the increased lattice volume as a result of the alloying elements [26]. Furthermore, when using the dependence of the plateau pressure of hydrogen sorption on the atomic radius factor for LaNi_5_ alloys [26], one could get an idea of the plateau pressure of the alloys in the present study, which is about 0.01 MPa for LaNi_4.3_Co_0.4_Al_0.3_. The data available in the literature related to the hydriding kinetics of similar alloys vary in a large range, from a few minutes to half an hour, to reach a full capacity of the alloy [14,28,29]. These differences were mainly due to the variances in the microstructure (defects, grain size) of the materials. In our study, as a result of using a similar alloys composition and identical preparation method, these differences were small, which allowed for correct comparison of the alloy hydrogen sorption properties.

### 3.3. Electrochemical Hydriding

**Figure 4:**
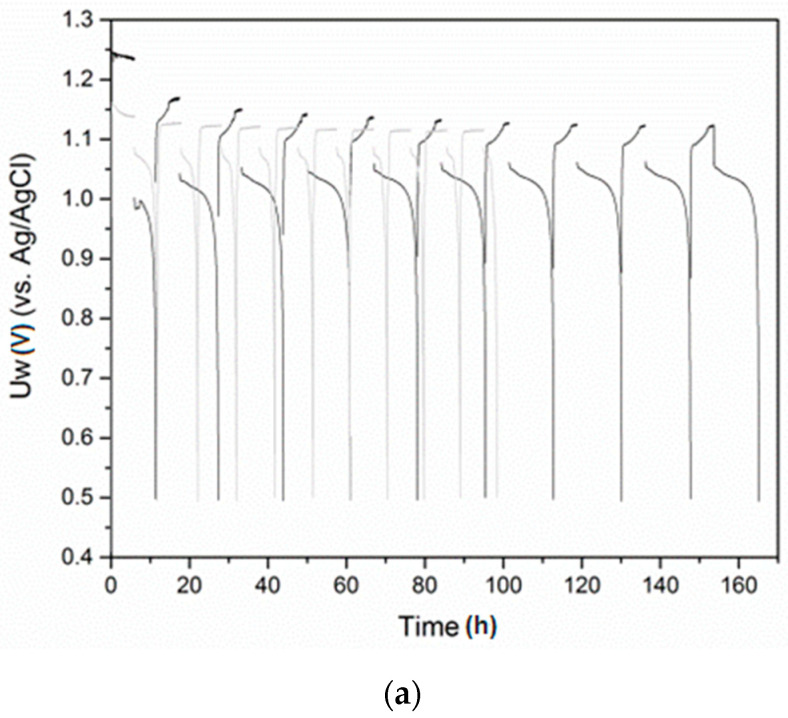

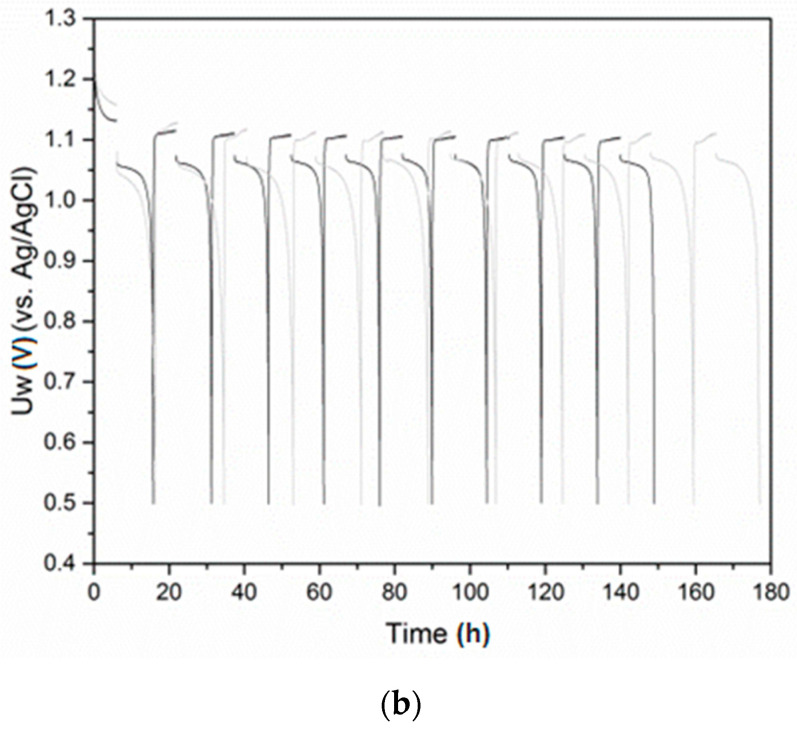
First 10 cycles for LaNi_5_ based alloys at current density of 5 mA during charge and 2 mA during the discharge. LaNi_4.3_Co_0.4_Al_0.3_ (black) and LaNi_5_ (gray) (**a**). LaNi_4.8_Sn_0.2_ (black) and LaNi_4.7_Sn_0.3_ (gray) (**b**).

**Figure 5:**
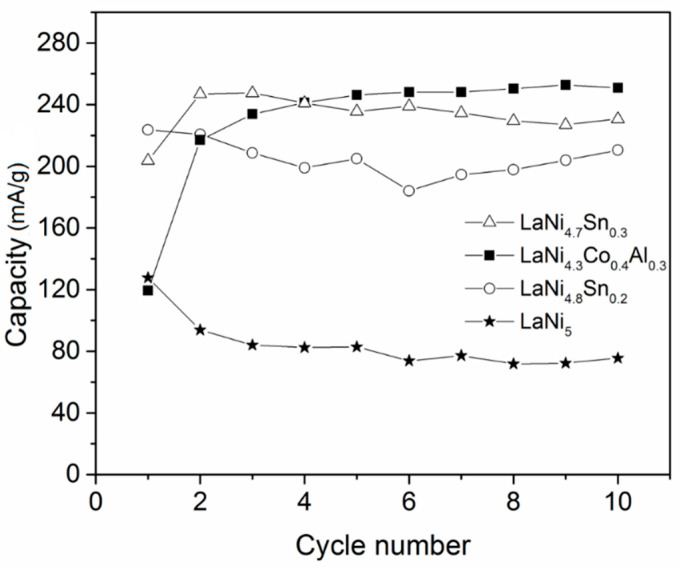
Discharge capacities vs. the cycle number for the alloys studied at a current density of 50 mA/g during the charge period and 20 mA/g during the discharge period.

The electrochemical hydrogen charge/discharge behavior of the alloys studied was also compared. Figure 4 shows the first 10 cycles at a current density of 50 mA/g during the charge process and 20 mA/g during the discharge process. The potential of the working electrode (Uw) as a function of time dependences showed that during the charge process, the metal hydride electrode potential for LaNi_4.3_Co_0.4_Al_0.3_ changed from 1.17 V to 1.13 V (vs. Ag/AgCl), approaching that of pure LaNi_5_. Similarly, the discharge curve of LaNi_4.3_Co_0.4_Al_0.3_ shifted to higher potential levels as a result of cycling. Both effects were due to the initial activation of the alloy. This was also clearly observed from the determined discharge capacities during cycling. For the Sn-containing alloys, both the charge and discharge potentials were close and correspond to those of LaNi_5_. The most evident and important difference however was associated with the hydrogen discharge capacity of the electrodes prepared from the different alloys, as can be seen in Figure 5. Among the studied alloys, only LaNi_4.3_Co_0.4_Al_0.3_ needed an initial (3–5 cycles) activation before reaching its maximum capacity of about 260 mA/g, which could be explained by an increase in the surface area of the electrode and surface activation during the initial charge/discharge cycles. Similar behavior for this alloy was also observed by Giza [21], as his study was limited to 10 charge/discharge cycles only. As can be seen further in this article, the high capacity achieved for LaNi_4.3_Co_0.4_Al_0.3_ remained stable long after 10 hydriding/dehydriding cycles were completed. The alloys with Sn also showed clearly improved capacity compared to pure LaNi_5_, as at this low discharge current density (20 mA/g), the sample with higher Sn content (LaNi_4.7_Sn_0.3_) slightly exceeded the capacity of LaNi_4.8_Sn_0.2_. The observed substantially higher discharge capacity of the Ni-substituted LaNi_5_ based alloys compared to pure LaNi_5_ could also be attributed to the increased crystal cell volume as a result of the alloying. It is worth noting that the electrochemical discharge capacities of the alloys corresponded as a trend (except for LaNi_4.7_Sn_0.3_) to the hydrogen absorption capacities measured in the hydrogen gas phase.

**Figure 6:**
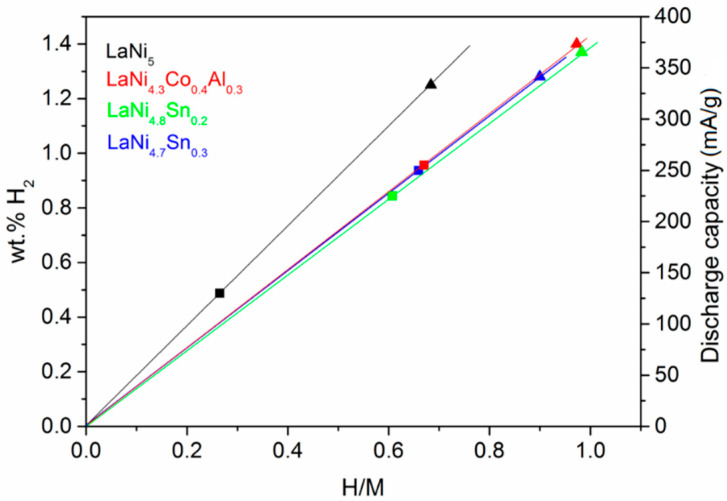
Hydrogen capacity as a function of the hydrogen content of the different alloys. The points correspond to the maximum alloys capacities measured by the two techniques—an electrochemical (■ in the corresponding color) and a hydrogen gas phase (▲).

Figure 6 summarizes the maximum hydrogen capacities measured by the two techniques used: the hydrogen gas phase and electrochemical hydriding. The lines corresponded to the theoretical capacities with a different hydrogen content for the alloys. It is noteworthy that the capacities determined by the gas phase hydriding were slightly higher than those that were determined electrochemically. Obviously, the electrochemical method fails to fully charge the alloys, which is not surprising due to the complex structure of the electrode, including electrode components that could partially block the surface of the active material particles.

**Figure 7:**
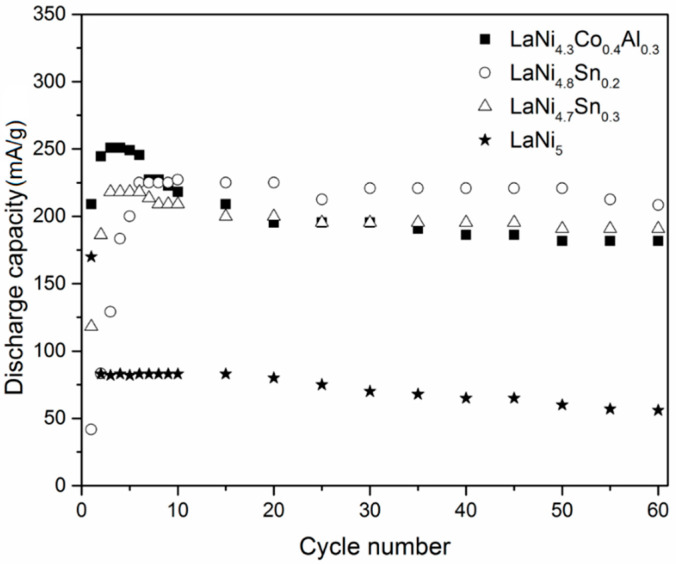
Discharge vs. cycle number curves for the alloys studied at a current density of 100 mA/g during charge and discharge.

Due to the practical importance of the LaNi_5_ based alloys used for the preparation of metal hydride electrodes in Ni–MH batteries, it was necessary to study their stability during multiple charge/discharge cycling. Figure 7 presents 60 charge/discharge cycles, measured at higher current densities (100 mA/g) of the charge and discharge process.

As can be seen from the cycling stability tests, all LaNi_5_ based alloys in this study revealed stable capacities during 60 cycles. They remained in the range of 175–200 mA/g for the alloyed LaNi_5_ based compounds. The alloys LaNi_4.3_Co_0.4_Al_0.3_ and LaNi_4.8_Sn_0.2_ showed the best cycling performance, retaining a relatively high discharge capacity of above 170 mA/g after 60 cycles. It is important to note that at higher current densities of charge and discharge (100 mA/g), the alloy with lower Sn content revealed a higher discharge capacity and a better cycle life compared to LaNi_4.7_Sn_0.3_ and the opposite was observed at a low current density (20 mA/g). This result, however, corresponds to a previous study on the electrochemical hydriding of LaNi_5−x_Sn_x_ (0 ≤ x ≤0.5), where it was shown that the relationship between the electrochemical discharge capacity and Sn content is not linear and strongly depends on the charge/discharge conditions [12].

These same (or very similar) discharge capacities measured at different current densities prove that a relatively high hydrogen diffusivity was present in the alloys studied, i.e., a clear indication of the lack of transport difficulties during hydrogen discharge. Independent measurements, based on a potentiostatic discharge of preliminary fully hydrogen-charged samples, were carried out to give additional evidence for the last observation and a quantitative assessment of hydrogen diffusivity.

**Figure 8:**
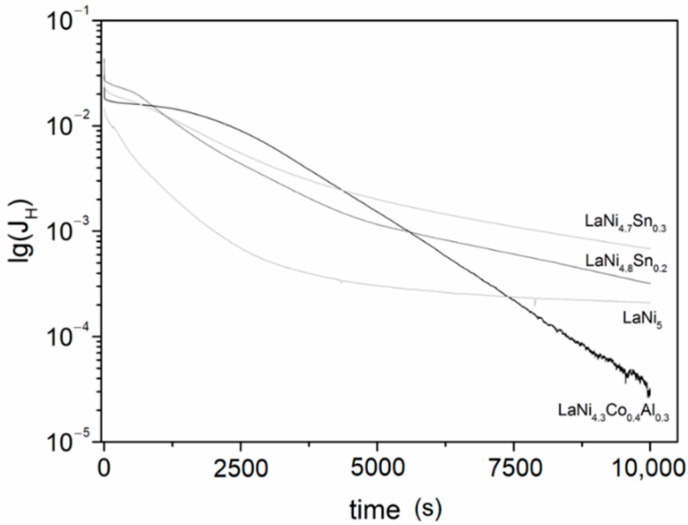
“lg(*J_H_*) vs. discharge time *t*” during potentiostatic discharge of LaNi_5_ type alloys.

Figure 8 compares hydrogen discharge curves of LaNi_5−x_M_x_ (M = Co, Al, Sn) alloys in coordinates “lg(*J_H_*) vs. discharge time *t*”. Thus, using the following approximation for the current density [30]:

(1) $$\mathrm{lg}\left(J_{H}\right)=\;\mathrm{lg}[+\frac{6FD_{H}}{da^{2}}\left(C_{o}-C_{s}\right)]-\;\frac{\pi^{2}D_{H}t}{2.303a^{2}}$$

the diffusion coefficients of hydrogen *D_H_* into the LaNi_5_ based alloys were determined. In this equation, *J_H_* is the current density, *t* is the discharge time, *a* is the size of the particles (average particle size, which is in the range of 20–25 μm for the alloys studied), d is the density of the alloy, *C_o_* is the initial hydrogen concentration and *C_s_* is the hydrogen concentration after the electrode is anodically biased. From the slope of the plot “lg(*J_H_*) vs. *t*”, Figure 8, hydrogen diffusion coefficients for the LaNi_5_ based alloys were determined, as seen in Table 2. The *D_H_* values estimated for pure LaNi_5_ and for the alloyed compounds were close to those obtained for similar alloys, which were usually in the range 10^−8^–10^−11^ cm^2^·s^−1^ [19,20,30,31,32,33]. It is worth mentioning that these values often significantly differed mainly due to the application of different methods for their assessment and because of the differences in the alloy microstructure (defects, grain size, etc.). In the present work, we compared the *D_H_* values of alloys with a similar microstructure that were obtained at the same experimental conditions of potentiostatic discharge, therefore the comparison could be considered as being reliable. Thus, it can be concluded that the diffusion coefficients of all studied alloys were higher than that of the pure LaNi_5_, as for LaNi_4.3_Co_0.4_Al_0.3_, the diffusion coefficient *D_H_* was one order of magnitude higher compared to LaNi_5_. The higher hydrogen diffusion coefficients of the alloys compared to the pure LaNi_5_ was in good agreement with the trend in the kinetic curves of hydrogen absorption as well.

The experimental results obtained in the study allowed a rough estimation of the time for complete hydrogenation of the alloys. Applying the Einstein diffusion equation (diffusion distance (x) x^2^ = 2·*D_H_*·*t*) and using the hydrogen diffusion coefficients *D_H_* (Table 2) and the average particle size (Figure 2), for LaNi_4.3_Co_0.4_Al_0.3_, this time was found to be about 5 min, while for LaNi_5_ it was more than 30 min. These values are higher than those experimentally determined by the gas phase absorption but confirmed the significantly improved hydrogen sorption kinetics of the Ni-substituted LaNi_5_.

## 4. Conclusions

Hydrogen storage capacity, hydriding kinetics, cycle stability and hydrogen diffusivity of three LaNi_5_ based alloys (LaNi_4.3_Co_0.4_Al_0.3_, LaNi_4.8_Sn_0.2_, LaNi_4.7_Sn_0.3_) were studied and compared to pure LaNi_5_. Among the alloys that were studied, LaNi_4.3_Co_0.4_Al_0.3_ showed the highest hydrogen gas phase capacity (1.4 wt.%) and electrochemical discharge capacity of 260 mA/g, which remained above 180 mA/g after 60 charge/discharge cycles at a current density of 100 mA/g. A significantly improved discharge capacity of LaNi_4.8_Sn_0.2_ was also observed, as this alloy revealed the highest cycling stability among the alloys studied with a capacity decrease of only 10% for 60 charge/discharge cycles. The hydrogen absorption kinetics of the Ni-substituted compositions was also found to be significantly improved compared to pure LaNi_5_. The determined hydrogen diffusion coefficients for all Ni-substituted alloys were higher than that of pure LaNi_5_; as for LaNi_4.3_Co_0.4_Al_0.3_, the hydrogen diffusion was an order of magnitude faster than in LaNi_5_. Good agreement was obtained between the hydrogen sorption kinetics determined by the gas phase analysis and that attained by electrochemical methods. In summary, among the LaNi_5_ based alloys in the present study, the best combination of hydrogen storage properties was found for LaNi_4.8_Sn_0.2_—high gas phase sorption capacity, electrochemical capacity and charge/discharge cycle life.

## Figures and Tables

**Table 1:** Experimentally determined lattice parameters, the crystal cell volume and the Atomic Radius Factor for LaNi_5_ based alloys.

Alloy	Lattice Parameters, Å		Cell Volume, Å^3^	Atomic Radius Factor (According to [18])
	a	c		
LaNi_5_	4.982	4.008	86.14	870
LaNi_4.8_Sn_0.2_	5.032	3.981	87.31	872
LaNi_4.7_Sn_0.3_	5.042	4.039	88.91	873
LaNi_4.3_Co_0.4_Al_0.3_	5.018	4.019	87.65	867

**Table 2:** Hydrogen diffusion coefficients for the LaNi_5_ based alloys.

**Alloy**	LaNi_5_	LaNi_4.8_Sn_0.2_	LaNi_4.7_Sn_0.3_	LaNi_4.3_Co_0.4_Al_0.3_
***D_H_* (cm^2^/s)**	2.8 × 10^−^^11^	8.8 × 10^−^^11^	1.1 × 10^−^^10^	3.3 × 10^−^^10^

## Data Availability

The data presented in this study are available on request from the corresponding author. The data are not publicly available due to the lack of such system within the university.
